# Natural Nanoparticles: A Particular Matter Inspired by Nature

**DOI:** 10.3390/antiox7010003

**Published:** 2017-12-29

**Authors:** Sharoon Griffin, Muhammad Irfan Masood, Muhammad Jawad Nasim, Muhammad Sarfraz, Azubuike Peter Ebokaiwe, Karl-Herbert Schäfer, Cornelia M. Keck, Claus Jacob

**Affiliations:** 1 Division of Bioorganic Chemistry, School of Pharmacy, Saarland University, D-66123 Saarbruecken, Germany; sharoon.griffin@uni-saarland.de (S.G.); irfan_masood_79@yahoo.com (M.I.M.); jawad.nasim@uni-saarland.de (M.J.N.); s8musarf@stud.uni-saarland.de (M.S.); 2 Institute of Pharmaceutics and Biopharmaceutics, Philipps University of Marburg, 35037 Marburg, Germany; cornelia.keck@pharmazie.uni-marburg.de; 3 Department of Biotechnology, University of Applied Sciences Kaiserslautern, 66482 Zweibruecken, Germany; karl-herbert.schaefer@hs-kl.de; 4 Department of Chemistry/Biochemistry and Molecular Biology, Federal University, Ndufu-Alike Ikwo, 482131 Ndufu-Alike, Nigeria; azubike.ebokaiwe@funai.edu.ng

**Keywords:** bioreduction, homogenization, microbes, nanoparticles, redox, selenium, sulfur, silver

## Abstract

During the last couple of decades, the rapidly advancing field of nanotechnology has produced a wide palette of nanomaterials, most of which are considered as “synthetic” and, among the wider public, are often met with a certain suspicion. Despite the technological sophistication behind many of these materials, “nano” does not always equate with “artificial”. Indeed, nature itself is an excellent nanotechnologist. It provides us with a range of fine particles, from inorganic ash, soot, sulfur and mineral particles found in the air or in wells, to sulfur and selenium nanoparticles produced by many bacteria and yeasts. These nanomaterials are entirely natural, and, not surprisingly, there is a growing interest in the development of natural nanoproducts, for instance in the emerging fields of phyto- and phyco-nanotechnology. This review will highlight some of the most recent—and sometimes unexpected—advances in this exciting and diverse field of research and development. Naturally occurring nanomaterials, artificially produced nanomaterials of natural products as well as naturally occurring or produced nanomaterials of natural products all show their own, particular chemical and physical properties, biological activities and promise for applications, especially in the fields of medicine, nutrition, cosmetics and agriculture. In the future, such natural nanoparticles will not only stimulate research and add a greener outlook to a traditionally high-tech field, they will also provide solutions—pardon—suspensions for a range of problems. Here, we may anticipate specific biogenic factories, valuable new materials based on waste, the effective removal of contaminants as part of nano-bioremediation, and the conversion of poorly soluble substances and materials to biologically available forms for practical uses.

## 1. Introduction

Today, nanotechnology and its diverse products are omnipresent and form an integral part of our products and lifestyle, from nanosilver in deodorants and nanoscopic particles with improved release properties in medicines all the way to “nanoimpregnations” of shower cabins, bath tubs and washing basins [1,2,3]. Whilst innovative materials containing particles with diameters in the one to one hundred nanometer range have emerged in many areas of our daily life, there has also been a feeling that such materials are “not quite natural”. Not surprisingly, therefore, the field of nano-toxicology more recently has attracted a particular interest—and there has also been mounting concern regarding a possible toxic impact on humans and contamination of the environment with nanomaterials [4].

**Figure 1:**
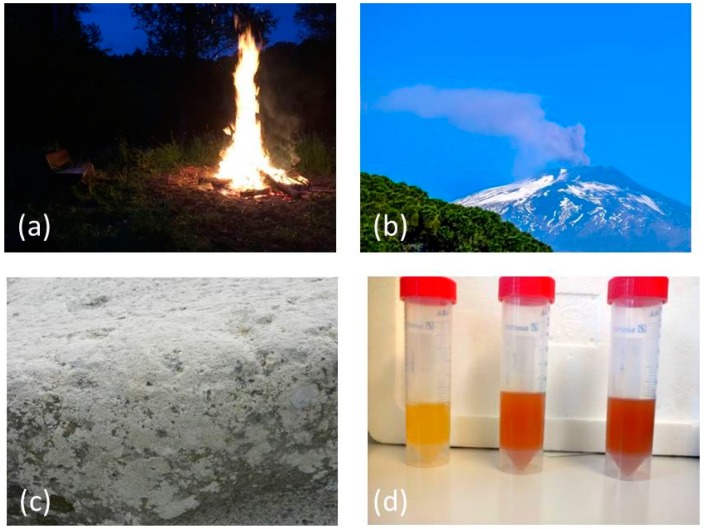
Nature itself is a skilled nanotechnologist. Microscopic and nanoscopic particles are formed, for instance, by combustion and are found: (**a**) near open fire; (**b**) as result of volcanic activity; (**c**) in form of precipitates; and (**d**) as bioreductively formed deposits of elements in certain bacteria. Photos provided by Marc Schäfer and Muhmmad Jawad Nasim.

This concern is certainly not entirely unjustified, as some dramatic examples, for instance asbestos (average diameter ranging from three to five micrometers) and other, air-bound fine particle matter, such as the PM_2.5_ fraction in exhaust gases, fumes and smoke illustrate [5,6,7]. Such critique, however, often ignores the fact that Nature itself is a skilled nanotechnologist, with numerous examples of common nanomaterials literally emanating from natural sources, such as volcanoes and mineral springs but also, in particular, from living organisms. Figure 1 provides a colorful reminder of such entirely natural sources of nanoscopic and microscopic particles. Indeed, life revolves around cells which themselves are microscopic in size (we do not account here for some rare and/or controversial nanobacteria) and metabolize molecules which are picoscopic, but also materials in between, which obviously are nanoscopic in their dimensions [8,9,10]. At the same time, nature also provides the inspiration and eventually also the ingredients—and even some of the methods—for natural nanomaterials.

**Figure 2:**
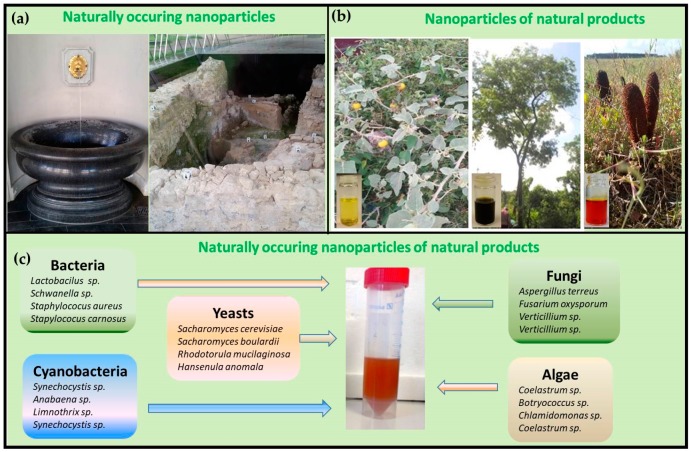
Examples of natural and biological materials which contain nanoscopic particles. (**a**) Naturally occurring nanoparticles of inorganic, elemental sulfur, for instance, are found at mineral wells rich in hydrogen sulfide, such as the Elisenbrunnen in Aachen. (**b**) In contrast, mechanically produced nanomaterials of natural products have been evaluated for medical and agricultural applications. (**c**) Eventually, there are also naturally produced nanomaterials of natural, biological products, such as nanoscopic particles of elemental selenium coated with microbial proteins which are formed by bioreductive or oxidative metabolism in bacteria and fungi.

Here, we will briefly consider the emerging field of natural nanoparticles. Before we start, we must, however, clarify what exactly is meant here. As any cunning linguist may have noted, the predicate “natural” may lead us into several directions. Indeed, there is a need for a distinction between nanomaterials which are, strictly speaking, natural, i.e., already present or formed in the environment without human intervention, and materials which are “nano” and also “bio”. In the second case, the predicate “natural” equates with “biological”, as in “natural products”, which refers primarily to biological substances or materials [11,12,13]. Figure 2 illustrates this divide and provides a few selected examples of particularly interesting natural nanoparticles, nanoparticles of natural products and, eventually, naturally produced nanoparticles of natural products which we will discuss in more detail in the following sections. From the outset, we should emphasize that the field of natural nanotechnology is wide and diverse. Since we are unable to grasp it in its entirety, we will focus on a few highlights which we consider especially instructive, for instance in the fields of natural product-based nutrition, cosmetics, medicine or eco-friendly, “green” agriculture.

## 2. Natural but Not Biological: The Free Flow of Inorganic Nanocomposites

Whilst hunting for nanoscopic materials in the environment, one soon realizes that a fair number of natural nanoparticles can be found outside the realm of life, for instance nanoscopic ash or soot particles as a result of volcanic activity, fires or other types of combustion. These particles are natural, yet usually not biological. Volcanic ash clouds contain a wide variety of polydisperse micro- and nanoparticles. These particles range from 100 to 200 nm in size and are chemically primarily composed of silicate and iron compounds. They are readily suspended in air and once inhaled may lead to serious respiratory disorders. Indeed, whilst particles of sizes in the lower micrometer range deposit in the upper respiratory tract, particles in the nanometer range penetrate deeply and deposit in tracheobronchial and alveolar regions where they can cause severe respiratory disorders [14]. “Carbon Nanotube” soot collected from the combustion of Texas Piǹon Pine, for instance, contains multi-walled carbon nanotubes of 15 to 70 nm in size. These carbon-based objects readily become airborne and pose severe health hazards to animals and the human population [15].

Fire, however, is only one chemical process in the inorganic sphere which eventually may result in nanoscopic particles. Precipitation, oxidation and, to a lesser extent, reduction are also well suited to turn naturally occurring, inorganic materials into nanoparticles (Table 1).

**Figure 3:**
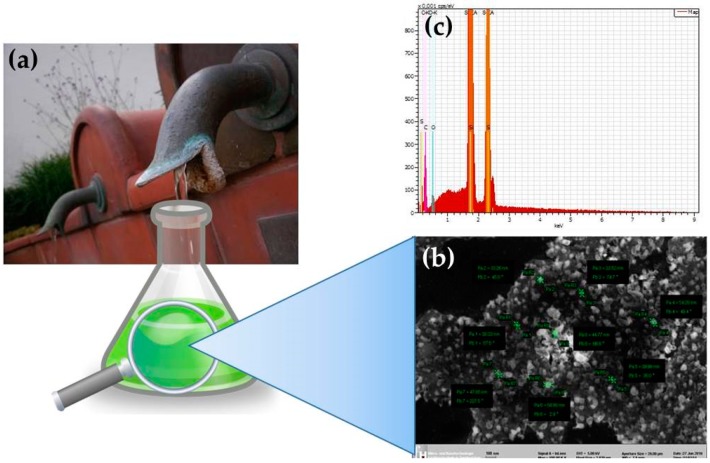
(**a**) The mineral wells in and near the town of Aachen in Germany are rich in sulfur, primarily in form of hydrogen sulfide (H_2_S). Solid deposits of inorganic matter can therefore be found, for instance, at the Marktbrunnen in Burtscheid (image kindly provided by Roman Leontiev). (**b**) A microscopic investigation at 10,000-fold magnification reveals numerous microscopic and sub-microscopic particles and irregular agglomerates in this kind of water which (**c**) according to Energy Dispersion X-ray spectroscopy (EDX) consist of primarily of calcium salts and elemental sulfur [42].

Amazingly, our drinking water, once considered under the microscope, is full of polydisperse nanoscopic as well as microscopic solid materials, of irregular shape and chemically based primarily on CaCO_3_ and CaSO_4_, often laced with other elements, such as iron oxides. Figure 3 provides an example of such deposits found near a mineral well in Aachen, Germany, and a microscopic view of the particles found in this water. Admittedly, such particles are of a rather poor quality and cannot be compared to the perfectly shaped, well-defined and homogeneous nanomaterials achieved by modern nanotechnological processes. Nonetheless, chemical, as well as physical processes, such as weathering, the slow precipitation of iron oxide particles but also dissolution and precipitation of carbonates under the influence of CO_2_ and the intermediate formation of hydrocarbonate (HCO_3_^−^), are able to generate such small-sized particle matter [55]. Abrasion, for instance, results in fine particle matter by scraping, cutting or grinding down larger lumps. There are numerous examples of natural nanomaterials formed this way, such as the CaSO_4_ and silicate particles in spring water [16]. Indeed, such inorganic particles recently have inspired colleagues to synthesize a wide range of similar particles based on naturally occurring materials, such as—refined—nanoparticles of Fe_3_O_4_ and MnO_2_ [56,57]. Concurrently, Nature, in cahoots with human activities, often unwillingly generates such “nanosized” particles from bulk materials, as the hot issue of microplastic in our oceans, fish and food highlights [58,59]. Nanoparticles generated by natural nanosizing are therefore not uncommon in our environment, and additional examples worth considering in earnest include the fine platinum particles released from millions of cars and their catalysts, as well as abrasion from tires, which are hardly “natural”, yet slowly, but continuously, affect our environment and eventually also our health [60,61].

Besides such “simple” physical and chemical events, there are similar, albeit more controlled particle generating processes, often based on spontaneous oxidation. One frequently observed example is the oxidation of hydrogen sulfide (H_2_S) gas or hydrogen sulfide ions (HS^−^) dissolved in waters of volcanic origin, such as volcanic lakes or mineral springs and wells which are common in many regions of the planet [62,63]. Here, the sulfide present in the water is oxidized by oxygen in the air to small particles of or containing elemental sulfur which can be found in the water itself, as in the Elisenbrunnen or Marktbrunnen in Aachen and, eventually, as part of larger sulfur deposits at or near the sulfide source. As before, Nature’s inorganic chemistry is not the best nanotechnologist, and those particles are of a rather poor quality, polydisperse and also not entirely pure either. Still, they are already quite well defined—at least regarding chemical composition—and represent an interesting nanomaterial which is formed entirely naturally and can be obtained in considerable amounts and concentrations. From the perspective of resources, the flow of sulfur nanoparticles from volcanic sources is both, sustainable and virtually free of any costs. In the future, such natural sulfur nanoparticles from mineral wells may well be “harvested” or employed directly, for instance as a substitute for colloidal sulfur in agriculture, or may require some “maturation” in form of spontaneous or controlled oxidation of the hydrogen sulfide contained within the water. Nonetheless, simple medical applications, for instance on skin, also appear feasible. Indeed, many of the mineral wells particularly rich in sulfide, such as Bad Nenndorf or Bad Wiessee in Germany, offer bathing—rather than drinking—as part of therapy [64].

From a more scientific perspective, there are also still issues in the water which may need to be resolved. The active ingredient(s) in these wells, for instance, may well be simple sulfide (HS^−^), as traditionally assumed, but also inorganic polysulfides (HS*_x_*^−^) or indeed elemental sulfur particles (S_8_) [42]. Curiously, as these three classes of species exhibit their own, characteristic physical and chemical properties and reactivities, for instance in the context of the “cellular thiolstat” [65]. These Reactive Sulfur Species (RSS) are also easily converted into each other in the presence of oxidants (such as air), reductants (such as glutathione) or even spontaneously by mutual interactions in form of an extensive sulfur-centered “redox scrambling” [66]. Similarly, selenite (SeO_3_^2−^) often occurs together with sulfur and, if reduced by H_2_S or HS^−^, is able to form a wide range of elemental sulfur, selenium and mixed selenosulfur nanoparticles. As in the case of the sulfur particles in mineral wells, such selenosulfur species are interesting from a more applied, biological point of view. Selenosulfur compounds are well established and widely known, for instance, as active ingredients of certain anti-dandruff shampoos and even feature in movies such as “Evolution” [67,68].

Most of these nanocomposites generated more or less randomly in the wild by crude chemical processes are of an equally crude morphology and complex chemical composition. Good quality nanoparticles of elemental sulfur, selenium and tellurium can be produced under more controlled conditions in the laboratory employing a very similar “chemistry”. The redox comproportionation of sulfide (HS^−^) and sulfite (SO_3_^2−^), for instance, results in nanoscopic sulfur monodisperse particles of almost uniform size and round shape, and with an average diameter of around 150 nm. Similarly, the reduction of selenite (SeO_3_^2−^) with the sulfur-containing amino acid l-cysteine yields spherical selenium nanoparticles with diameters in the range of 50 nm. Reduction of tellurite (TeO_3_^2−^) with hydrazine (N_2_H_4_) even enables the generation of tellurium particles in the form of nanoscopic needles [69].

Inspiration for this kind of simple redox chemistry does not stop at the chalcogens. There are many other examples of spontaneous chemical (redox) transformations which eventually lead to small particles, such as the reduction of silver (Ag^+^) or gold (Au^3+^) cations to elemental silver or gold particles, respectively. In the following sections, we will therefore consider such reduction and oxidation reactions also in the context of other natural, biological agents.

## 3. Bioreductive Formation of Nanoparticles

**Figure 4:**
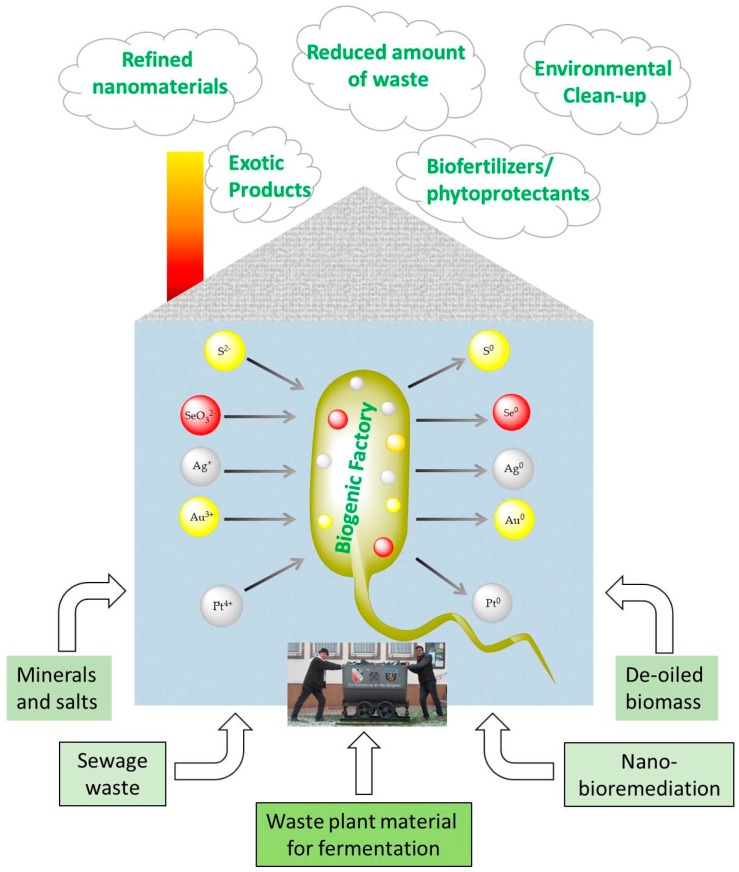
Schematic illustration of the biogenic factory which is able to turn biological substances, extracts, plants, algae and even waste biomass into amazing new products and nanomaterials. A particular interest resides on the added value resulting from the use and “up-cycling” of by-products and waste, such as de-oiled herbs, spent grains and coffee grounds, as these materials initially are not only food-grade but otherwise would go to waste and hence impact negatively on the environment. Photo taken at Hassel (Saar) and kindly provided by Elizabeth Jacob.

Whilst volcanoes and mineral springs rely on simple chemical transformations to generate nanoparticles, living cells can recruit an entire arsenal of biotransformations to eventually produce such composites. Indeed, the living cell is mostly dealing with “nanotechnology”, i.e., with objects of a nanoscopic size. Just to get a feeling for dimensions: A strand of DNA is 2.5 nm in diameter, a typical virus is around 100 nm wide and a typical bacterium is ten times bigger, i.e., in the range of 1–3 µm. Mammalian and plant cells are comparably large, occasionally reaching 50 µm in diameter [70,71]. It is therefore not surprising that cells, and here microbial cells in particular, engage in some sort of “nanotechnology”. Still, in the context of nanoparticles, there is one major caveat: Living cells normally do not prefer “the solid state”, as any deposits formed intracellularly may stress and eventually kill them in a suicide-like process. The expulsion of such particles requires a more sophisticated machinery, effort and energy for excretion. Hence the formation of deposits inside cells is not that common—but also not entirely unknown either. When exposed to inorganic salts (e.g., containing S^2−^, SeO_3_^2−^, Ag^+^ and Au^3+^), certain bacteria such as *Pseudomonas aeruginosa*, *Thiobacillus, Serratia*, and *Stenotrophomonas* species employ a reductive or oxidizing pathway of detoxification which eventually leads to the formation of elemental particles [72,73,74,75,76,77]. Such processes are rather well established and studied in the context of sulfur and selenium as well as silver nanoparticles, but also seem to apply to nanoparticles of gold and even platinum [78]. The particles produced by such biogenic factories are often of a surprisingly good quality, for instance small spherical shapes of an almost uniform size (Figure 4).

Not surprisingly, such biological processes may be exploited in practice to produce such particles of good quality and yield. This is the case, for instance, for selenium nanoparticles in dairy products produced by bacteria such as *Shewanella* sp. and *Lactobacillus* sp. Here, microorganisms which ferment the milk are also able to reduce selenite to elemental selenium for an additional “kick” [79,80]. Such processes are also of interest in the context of bioremediation and decontamination of soils enriched in certain toxic metals or semi-metals. Indeed, the removal of environmental contaminants (such as heavy metals, organic and inorganic pollutants) from contaminated sites using nanoparticles or nanomaterials formed by or in plants, fungi and bacteria with the assistance of nanotechnology, often referred to as nanobioremediation (NBR), is an emerging, environmentally friendly and economical alternative to traditional chemical methods [81,82]. Here, the three main strategies of modern bioremediation include the use of plants, microbes and isolated enzymes, for instance, laccase or nitrate reductase [83,84].

Whilst bioremediation is clearly an emerging topic related to microbially formed natural nanoparticles, it aims primarily at the removal of contaminants. Even so, there is also a more positive side to this approach. Here, the nanoparticles generated by such organisms are no longer seen as contaminants but actually as valuable nanomaterials of a more or less natural origin (Figure 4). Within this context, several of these bioreductively formed “natural” nanoparticles have been explored recently with sight on potential medical and agricultural applications [85,86,87,88].

It is possible, for instance, to recruit harmless microorganisms, such as *Saccharomyces cerevisiae* and *Staphylococcus carnosus* to generate fairly homogeneous selenium nanoparticles from SeO_3_^2−^ with average diameters of 60 nm and 80 nm, respectively [89,90]. These particles can be harvested from the yeasts and bacteria after lysis of the cell. The authors of such studies have speculated about possible applications as food supplements and possibly as antimicrobial agents as some of these particles exhibit a certain antimicrobial activity [89,90,91]. In the field of agriculture, possible applications have even more facets, and a possible “hat trick” of simultaneously enriching the soil with selenium for fortified food products, of providing plants with elements for their natural defense systems and of eradicating plant pathogens seems feasible [89].

Within this context, one needs to emphasize that such naturally generated particles are not comparable to industrially generated materials. They are not “chemically pure”, and usually also contain a “natural” coating of proteins whose composition is a reflection of the yeasts or bacteria they have been produced in. Hence the biological activity of such natural particles may stem from the bulk material of the particle itself, such as selenium, from other compounds trapped or contained within the particle, and also from the coating, which is often rich in proteins [89,92,93,94]. In such cases an extensive “intracellular diagnostics” is required to elucidate the exact target(s) and precise mode(s) of action [95].

Eventually, one may envisage an elegant process by which bacteria are grown on contaminated soils, and by remediating those soils produce well-defined nanoparticles which may be harvested and used in medicine, agriculture or other suitable applications. The resulting benefits of such an approach may be substantial—and are not far-fetched either, as relevant contaminants, such as heavy metals, often also represent the basis of particularly interesting particles.

As these strategies traditionally have focused primarily on the production of nanomaterials, the fate of the microorganisms involved usually has been of minor importance. Still, there may be some additional benefits, especially in the context of pathogenic fungi and bacteria. Several studies have demonstrated that the formation of nanosized materials by and inside pathogenic bacteria is an effective instrument to destroy those organisms. It has been noticed, for instance, that pathogenic strains of *Staphyllococcus aureus*, such as HEMSA and HEMSA 5M, reduce SeO_3_^2−^ to elemental selenium when confronted with exceptionally high concentrations of this anion (around 2 mM) in an apparent attempt to deal with this exposure [96]. Eventually, this protective strategy fails and the deposits of selenium formed inside the bacteria kill these cells. This kind of “suicidal natural nanotechnology” is found among many bacteria and fungi, including pathogenic ones [89]. It partially explains in part the antimicrobial action often associated with SeO_3_^2−^ and SeO_4_^2−^ and other Reactive Selenium Species (RSeS), as well as with TeO_3_^2−^ and TeO_4_^2−^ [89]. Such activities may be specific for certain organisms, endowing these agents and associated processes with certain “sensor/effector” properties. In the future, this kind of natural nanotechnology therefore may provide an interesting avenue to compromise, weaken, damage or perhaps even kill such pathogenic organisms [89].

## 4. Redox Chemistry with Natural Products

Just as many bacteria and fungi are able to produce nanoparticles of fairly good quality, this approach requires a certain effort in form of culturing and harvesting. It frequently also results in contamination with microbial biomolecules. Not surprisingly, alternative strategies have been developed which employ specific, isolated cellular components instead of whole cells to achieve the kind of—mostly bioreductive—chemistry which is usually required for the biological production of nanoparticles. As mentioned above, enzymes, such as laccase and nitrate reductase, are already employed in NBR, and similar avenues, based on isolated enzymes and simple natural reductants (or oxidants) have recently been explored as a means to generate nanoparticles [97,98,99,100]. Ascorbic acid, l-cysteine, reduced glutathione (GSH), flavonoids and a couple of other natural reducing agents are rather abundant in Nature and easy to obtain. Not surprisingly, these agents have been investigated already to produce nanoparticles of sulfur, selenium and silver, to name just a few [101,102,103,104,105]. Other redox active secondary metabolites, such as terpeniods (e.g., eugenol), flavonoids (e.g., luteolin and quercetin), sugars (e.g., glucose and sucrose) and certain amino acids (e.g., aspartate) have also been employed successfully to generate metal nanoparticles [101]. Besides simple plant metabolites, peptides have been considered, for example oligopeptides containing tryptophan residues. These peptides reduce metal ions to peptide-functionalized silver and gold nanoparticles [106]. Larger molecules, including redox active proteins, can also—chemically—produce nanoparticles, for instance particles of elemental platinum [107]. There are also reports that proteins from natural sources, such as whole cow milk, reduce metal cations, and generate, for instance, good quality nanoparticles of silver [108].

These few selected examples demarcate a particularly promising field of natural, biological nanotechnology, whereby isolated natural compounds, mixtures or even entire articles, such as whole milk, are used to produce nanomaterials. In practice, Nature provides a plethora of such reducing agents in form of compounds, peptides, proteins and enzymes. Indeed, certain microorganisms, plants and plant extracts are rich in antioxidants, with millimolar concentrations of ascorbic acid and thiols present therein [109,110,111,112]. As any lover of marmite will know, such extracts can be acquired rather easily, often as left-overs or by-products, such as yeast extracts from breweries. From an ecological and economical perspective, extracts are often superior to whole organisms and plants but also to isolated and extensively purified substances. Not surprisingly, therefore, such extracts are not only interesting from the prospect of being natural and fit for human consumption, but also since they are widely available and cheap [113,114]. Within this context, one rather noteworthy study has employed aqueous extracts of the fungus *Amylomyces rouxii* (strain KSU-09 isolated from the roots of *Phoenix dactylifera*) to generate silver nanoparticles [115].

**Figure 5:**
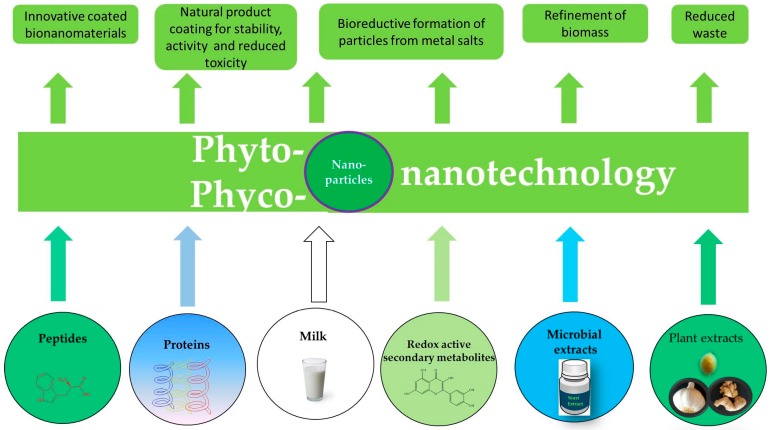
The emerging field of phyto-nanotechnology employs isolated biological components and substances to form, modify or coat nanoparticles. These particles often exhibit interesting properties, such as pronounced biological activity, and may therefore be employed in medicine or agriculture. Phyto-nanotechnology also offers new and innovative uses for plant materials and biomass, which otherwise may have been wasted. Here, the field of phyco-nanotechnology, which is centered around algae, for many biological, manufacturing, ecological and economical reasons today represents a particularly interesting area of research and development.

In the context of plants, in particular, extracts are common and readily available. Not surprisingly, aqueous extracts of *Nelumbo nucifera* (root), *Embelia ribes* (seed), *Rosmarinus officinalis, Ocimum basilicum*, *Petroselinum crispum* (leaf and root), *Citrus limon* (peel), *Vitis vinifera* (peel), *Cucumis sativus* (peel), *Mimusops elengi* Linn. (leaf), *Acalypha indica* (leaf), *Zingiber officinalis* and *Capsicum frutescens*, among others, are frequently used to produce nanoparticles of Ag, Au, Fe_3_O_4_ and ZnO [116,117,118,119,120,121,122,123,124]. Indeed, this emerging field of “phyto-nanotechnology” provides numerous advantages (Figure 5). The materials employed, such as extracts of herbs, are often available as cheap by-products, yet still rich in active ingredients, and therefore of value for further processing. One should also remember that some of these plant products are “food grade”, and hence entail possible applications in nutrition and cosmetics [125]. Together with other “readily available” biomass, such as microalgae, such materials are well suited for the controlled synthesis of good quality nanoparticles [126]. Indeed, the emerging field of “phyco-nanotechnology” relies explicitly on algae for bio-nanomanufacture as these organisms are not only highly interesting from a scientific point of view, but also readily available, easy to culture and environmentally friendly to use [127].

Besides these more obvious applications of extracts and by-products in phyto-nanotechnology, one should also briefly mention two additional applications in this field. One is the use of such “waste” biomass as feedstock for bacteria and fungi able to generate nanoparticles in vivo [101,128]. In this case, the biomass is not used directly as reducing material, as above, but rather indirectly—and probably more extensively—to promote the growth of suitable bacteria able to perform this kind of bioreduction. The second application concerns the coating of nanoparticles. As mentioned above, nanomaterials produced by bacteria are often coated with proteins, and this coating may endow such particles with improved stability, further features and especially also additional biological activities. It is therefore not surprising that natural substances, such as extracts of Darjeeling tea, have been investigated as coatings for silver nanoparticles to provide stability against agglomeration and also to reduce toxicity [129].

In general, these materials—literally—provide a fertile ground for future research and development, especially in the context of turning biomass waste into (nanomaterial) value (Figure 5).

## 5. Milling Vanilla

Thus far, natural products have been employed primarily to generate, cover or coat nanoparticles. This raises the question if such biological materials themselves may not be converted into nanoparticles. Similar to naturally occurring abrasion mentioned earlier, methods such as grinding, milling and (high pressure) homogenization provide a wide and colorful arsenal of methods able to “mill down” almost any material, including chemical elements in their solid state, sparingly soluble food supplements and medications, and, actually, also plant parts and even entire trees [130]. The resulting particles of such natural products are of a unique nature, as they are still natural products, yet have been transformed into an unusual, unnatural size and shape.

It is therefore hardly surprising that many natural products have been nanosized (or “nanonized”) during the last couple of years. Antioxidants such as rutin, for instance, have been turned into so-called “nanocrystals” using an eloquent technique which involves wet bead milling (WBM) and high-pressure homogenization (HPH) [131,132]. Here, nanotechnology can be used to produce nanoparticles with a dramatically improved solubility, excellent release kinetics and hence a good bioavailability and biological activity. This approach is particularly attractive in the field of—often sparingly soluble—antioxidants and plant products rich in such antioxidants, i.e., substances and materials which originally have poor release kinetics on the lipid/aqueous surface of the skin but thanks to the new technology can nowadays be used easily, for instance in cosmetics.

**Figure 6:**
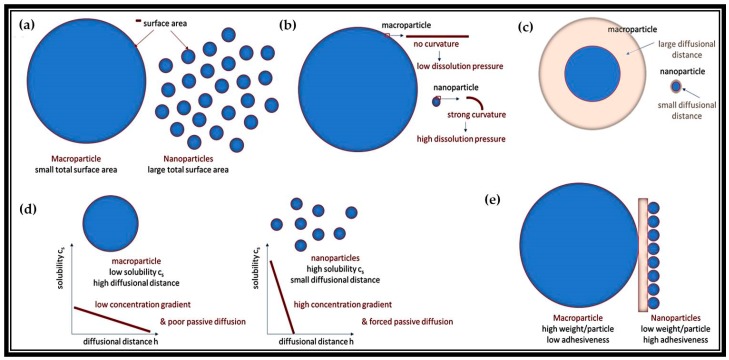
Properties of nanoparticles: (**a**) increased rate of dissolution; (**b**) enhanced saturation solubility; (**c**) decreased diffusional distance; (**d**) higher concentration gradient; and (**e**) improved adhesiveness.

The basic physical principles behind this approach of nanosizing natural products are illustrated in Figure 6. Indeed, the principle of nanosizing coarse materials to improve their biological activity is very simple and is mainly based on the Noyes–Whitney equation, one of the major equations in biopharmacy (Equation (1)).

(1) $$\frac{dc}{dt}=D\cdot A\cdot \frac{\left(c_{s}-c_{0}\right)}{h}$$

where *dc*/*dt* is the dissolution rate, *D* the diffusion coefficient, *A* the total surface area of the particles, *c_s_* the saturation solubility of the active ingredient, *c*_0_ the concentration of dissolved active ingredient in the solvent and *h* the diffusional distance.

When nanosizing coarse material the rate of dissolution *dc*/*dt* increases, because the total surface area of the particles involved increases (Figure 6a). In this case, the saturation solubility increases due to a higher dissolution pressure, which is explained by a higher curvature of the particles (Kelvin equation, Figure 6b) and the diffusional distance is also decreased (Prantl equation, Figure 6c). Eventually, nanosizing leads to a significant increase in the overall velocity of dissolution, which is especially interesting if active ingredients dissolve slowly or are even poorly soluble in water. Furthermore, nanosizing improves the bioactivity of poorly soluble active ingredients. As a result of the increase in solubility the concentration gradient, when compared to larger sized materials, is increased [133]. If the active ingredient is taken up by the body (or the plant) via passive diffusion, the concentration gradient is the driving force for uptake or permeation. Hence the higher the concentration gradient, the faster and more efficient the uptake will be (Figure 6d). Eventually, the smaller the size of a particle, the faster it will dissolve and the higher its bioactivity will be [134].

There are also additional benefits. Nanosized materials possess a much higher adhesiveness to surfaces than coarse materials. This is due to the much larger surface to volume ratio of nanoparticles, which translates into considerably more attaching points per volume and therefore to less forces needed to stick to a surface (Figure 6e). Hence, after administration or application, nanoparticles tend to adhere much tighter and longer to surfaces, the time to dissolve and to penetrate is prolonged, therefore further increasing the bioactivity of active ingredients. Due to these superior features and the ease of production, nanosizing, i.e., the production of nanocrystals, has become a major formulation principle in pharmaceutics to improve the bioactivity of pharmaceutically active ingredients [135,136,137].

In the field of natural products, nanosizing has opened up a promising avenue to augment further their potential. It is possible, for instance, to convert simple, intrinsically “insoluble” materials, such as elemental sulfur, selenium and tellurium—and, of course, many of the other solid elements of the Periodic Table—into nanosuspenions with interesting biological activities [69]. In the case of the three chalcogens, a pronounced biological activity, for instance against *Steinernema feltiae*, *Escherichia coli* and *Saccaromyces cerevisiae* has been observed which compares well with the one of the corresponding elemental particles obtained by redox chemistry or bioreduction in *S. carnosus* [89].

**Figure 7:**
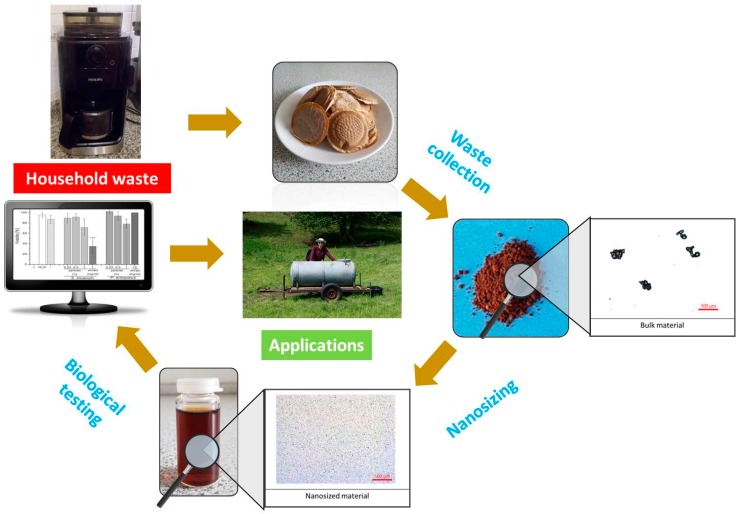
A schematic overview of theutilization of nanosizing techniques for turning waste into value.

Nanosizing chemically pure substances or mixtures is comparably straight forward, yet the matter literally becomes more complicated once natural samples such as barks, shells, seeds or even dried fruits, roots or entire plants are milled and homogenized. Here, specific techniques need to be developed and applied. It may appear straight forward to mill down a shell of a walnut or some commercially available grape seed flour, even so our experience tells us that nanosizing a dried tomato plant or spent coffee ground is considerably more challenging. Specific protocols with several steps may be required, from drying and freeze-drying to defatting and pre-milling. Still, some plants, such as the Maltese mushroom *Cynomorium coccinem* L., a parasitic plant devoid of any chlorophyll, can be obtained, freeze-dried, milled and homogenized to fairly stable, low polydispersity and uniform particles with average diameters of around 400 nm without encountering any unsurmountable problems [138]. Some of these milled down plant materials have been evaluated already as potential food supplements and even as natural medicines and antimicrobial agents [130]. In the medium term, nanoparticles of natural products may be employed in the fields of nutrition, medicine, cosmetics or, in the case of large scale manufacture, in “green” agriculture (Figure 7).

The activities observed for those nanosized materials are often promising, yet there is a fine balance of arguments which needs to be considered. On a positive note, nanosizing an herb or medical plant is comparably straightforward and considerably easier than extraction, purification and formulation of the active ingredient(s) contained therein. It also produces no or little waste. Furthermore, the nanoparticles essentially are still “natural”, at least as far as their chemical composition is concerned, contain all the ingredients of the plant, have not undergone any extensive modifications and, notably, have not been treated with any organic solvents. Ideally, they even represent a natural slow release system of bioavailable and biologically active ingredients. In the case of HPH, such materials initially are also sterile and, as more recent studies have confirmed, can also be lyophilized and resuspended without loss of physical properties or activity once a simple stabilizer such as mannitol is added [139,140,141].

Eventually, some caution is required as such materials are intrinsically ill defined chemically, often contain fibrous materials, are prone to fouling if contaminated with microorganisms and also require certain stabilizers so not to aggregate in nanosuspension or as a result of freeze-drying. In analogy to the 1980s German pop band “Milli Vanilli”, milling vanilla is clearly exciting, fancy, hot and full of potential, yet some care must be taken and there is still considerable need for further investigation and improvement, especially once the power fails and the chips are down [142].

## 6. Conclusions

**Figure 8:**
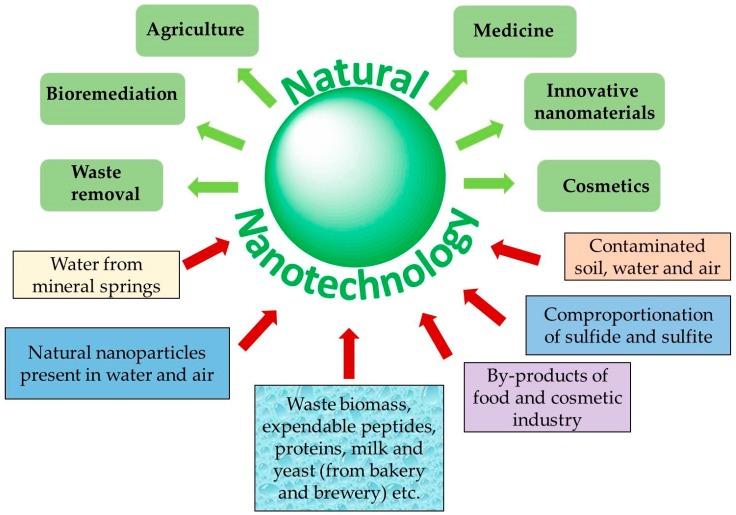
Natural nanotechnology with its various inorganic and biological aspects provides a wide range of opportunities and applications, not only in medicine and cosmetics, but also in less obvious areas such as agriculture and waste removal.

The previous sections have highlighted just a few selected recent developments at the interface of nanotechnology and natural products research. We have seen that nature itself is well suited to produce a repertoire of nanomaterials by processes such as combustion, abrasion, precipitation and oxidation, and, if the biosphere is included, by bioreduction and related processes. Some of the natural nanoparticles obtained in this way may be useful in medicine, agriculture or other fields of technology and engineering. Not surprisingly, this has stimulated research into these native materials and processes, and has also inspired strategies to generate biological and biologically active materials using similar materials and methods. It is now time to take stock and to anticipate some of the most exciting developments which the next couple of years may bring (Figure 8).

First, it seems plausible that some freely flowing inorganic substances, such as H_2_S, which hitherto have “only” been used in spa towns to pickle and macerate affluent pensioners or have been wasted entirely, may be reconsidered as valuable precursors of fine chemicals, including certain nanoparticles. Here, some “hat tricks” may also be feasible, such as the reaction of H_2_S from mineral springs with fumes rich in SO_2_ as part of an elegant sulfur redox comproportionation, or a reaction of H_2_S with NO_2_. Such “waste chemistry” may not only be employed to produce the desired nanoparticles—in this case of sulfur, but also to detoxify two individual environmental hazards simultaneously [42]. Those ideas are still speculative, however, early studies into this direction are marred by issues, such as adequate concentrations and how to bring the hazards—literally—under one roof. It seems to us that crucial but manageable research is required to define the correct ingredients and conditions for such manufacture and large-scale production of particles of sulfur and related elements, such as selenium.

Secondly, natural nanotechnology employing organisms such as yeasts and certain harmless bacteria, but also isolated enzymes, may in future be recruited to generate a variety of particular particle matter, starting with selenium and embracing large parts of the Periodic Table, but also other inorganic materials, such as insoluble metal oxides. Indeed, it seems today that a wide palette of insoluble matter may be generated inside bacteria. Whilst traditionally harvesting of such particles has involved lysis of cells, some organisms also release their particles into the supernatant, as has been demonstrated for resveratrol-conjugated gold nanoparticles produced by the *Delftia* sp. strain KCM-006 [143]. Such in vivo generation of nanoparticles may provide further impetus for bioremediation and inspire new avenues to tackle some pathogenic organisms unable to release their particles with undesired intracellular deposits.

Thirdly, the use of certain plant products, such as de-oiled herbs, as reagents in nano-manufacture may be considered more widely, especially in the context of waste management and “up-cycling”. Here, plant waste from harvests or processes such as baking or brewing may provide an interesting alternative to pure chemicals, as they are natural and readily available in large quantities and at low cost. As mentioned already, these materials may either be employed directly as reducing agents or coatings or, more indirectly, as feedstock for bacteria able to generate nanoparticles via a bioreductive avenue. From an ecological perspective, it would be especially intriguing to employ nanosized waste as feedstock for bacteria naturally producing natural nanoparticles.

Fourthly, milling and homogenization of plants may unlock a whole treasure chest of new products, as it can be employed to render hitherto insoluble materials into nanosuspensions with interesting release properties. Many of these products originate in agriculture and, once processed, may be used there as well, hence providing the basis for interesting production and application cycles. This field is still in its early stages of research and development and most certainly will lead to many obstacles and pitfalls, but also to some truly innovative ideas, methods and products.

Eventually, we are likely to witness a rapidly growing interest in various fields of bio-nanotechnology, such as phyto- and phyco-nanotechnology, not only with sight on product development, but also in many areas of basic research which accompany such developments. In the longer run, it may even be possible to explore some of these leads to generate nanoparticles of natural products, such as active ingredients of plants, using natural processes, including bacterial or fungal fermentation, in vitro bioreduction or abrasion. Here, the two meanings of natural, i.e., in form of the material or as part of the method, may eventually meet and merge. Most of this is obviously still speculative today, and time will tell which of these leads are green, fruitful inspirations and which are more the kind of red herring which will stay in the fishbowl of the laboratory.

## Figures and Tables

**Table 1:** Selected examples of naturally occurring inorganic micro- and nanoparticles frequently found in our environment and associated with certain possible applications or biological implications. Please note that the practical applications mentioned usually rely on refined materials of particularly good particle quality and purity and not, or not yet, on the crude naturally occurring materials of similar constitution and composition.

Name	Chemical Formulae and Symbols	Natural Occurrence	Practical Implications (of Similar, Refined Materials)
Calcium Carbonate	CaCO_3_	natural surface water [16]	industry, biotechnology, cancer therapy, drug delivery, plant nutrition and promotion of plant defense against pests [17,18,19,20]
Alumina	Al_2_O_3_		desalination and defluorination of water [21,22,23]
Silicate	SiO_4_^4−^		drug carrier and catalytic applications [24,25]
Silica	SiO_2_	volcanic eruptions [26]	food additive, anti-caking agent, ultraviolet antireflection coating, cellular imaging and biomedical applications [27,28,29,30]
Bassanite (Calcium Sulfate)	CaSO_4_	sea water [31]	bone regeneration [32]
Iron Oxide	Fe_3_O_4_	iceberg-hosted sediments [33]	medical diagnostics, controlled drug release, hyperthermia, biosensors, supercapacitor applications [34,35,36,37]
Manganese oxide	MnO_2_	umber [38]	imaging, remediation of contaminated soil and ground water, catalysis [39,40,41]
Sulfur	S	mineral wells [42]	medical applications, (antimicrobial, cytotoxic), fertilizers, fiber industry [43]
Soot (in the form of carbon)	C	atmospheric particulate matter	composite reinforcements, nano-reactors, chemical sensors, gas adsorbents, catalyst supports, templates, actuators, probes, nano-pipes [44,45]
Silver	Ag	aquatic environment [46,47]	antimicrobial properties, nano-functionalized plastics, paints, food containers, domestic appliances, textiles, medical products and cosmetics [46,48]
Gold	Au	ore deposits [49]	biosensorics, immunoassays, medical applications and laser phototherapy of tumors [50]
Platinum	Pt	automobile exhausts [51]	biomedical applications, nano-biomedicine, catalytic and thermal applications [52,53,54]
